# Evaluation of assistance systems allowing older drivers to intercept moving inter-vehicular space

**DOI:** 10.3389/fpsyg.2023.1244646

**Published:** 2023-10-24

**Authors:** Lola Tran Van, Catherine Berthelon, Jordan Navarro, Cédric Goulon, Nicolas Mascret, Gilles Montagne

**Affiliations:** ^1^Aix Marseille Univ, CNRS, ISM, Marseille, France; ^2^Université Gustave Eiffel, Salon-de-Provence, France; ^3^Université Lumière Lyon 2, Laboratoire d’Etude des Mécanismes Cognitifs, Lyon, France

**Keywords:** road safety, ADAS, older drivers, acceptance, perception-action coupling

## Abstract

**Introduction:**

The objective of the present study was to test two Advanced Driving Assistance Systems (ADAS) designed to help older drivers to intercept a moving inter-vehicular space.

**Method:**

Older and younger drivers were asked to intercept a moving inter-vehicular space within a train of vehicles in a driving simulator. Three ADAS conditions (No-ADAS, Head Down, Head Up) as well as five distinct speed regulation conditions were tested. Vehicle trajectory, gaze behavior and acceptance were analyzed.

**Results:**

Our results reveal that the ADAS tested make it possible to perform the interception task but also to reduce the variability of the behavior produced. They also indicate that the location of the augmented information provided by the ADAS directly impacts the information-gathering strategy implemented. Finally, whereas younger divers reported mixed levels of ADAS acceptance, older drivers reported a good level of acceptance.

**Discussion:**

All these results could be particularly useful with a view of designing ADAS for older drivers.

## 1. Introduction

In 2020, the European Road Safety Observatory^1^ reported that 18,800 people were killed (and more than 500,000 injured) in road accidents in the European Union; maneuvers performed when approaching an intersection account for 19% of road fatalities, or 3,570 deaths.

The factors associated with road accidents at intersections have been identified in the literature. They concern first the complexity of the maneuvers to be produced, which require the performance of several tasks simultaneously. The driver must not only identify the type of intersection to cross (T, Y, number of branches, etc.), but also make the right decision (e.g., to cross or not to cross) and regulate speed accordingly based on the information gathered from the driving environment. Second, the study by Caird and Hancock (2002) revealed that this type of accident is also related to the characteristics of the drivers.

Older drivers are a particularly vulnerable population. In 2020, adults aged over 65 were involved in 28% of traffic fatalities (all maneuvers combined) in the European Union (European Commission, 2022). The epidemiological data also reveal the severity of accidents in which older drivers are involved. The propensity of older people to be prone to accidents is even higher when driving conditions become difficult (Cerelli, 1995; Chandraratna and Stamatiadis, 2003; Mayhew et al., 2006). This is caused by visual, cognitive and physical alterations that are generally associated with ageing (Case et al., 1970; Ball et al., 1993, 1998). For example, older drivers are less efficient than middle-aged drivers to spot hazards in peripheral vision (Yamani et al., 2016). These difficulties can be partly attributed to the limitations of their useful field of view (Bromberg et al., 2012; Douissembekov et al., 2015). Poorer parking maneuvers were observed with a narrower useful field of view through ageing for instance (Douissembekov et al., 2015). Because the EU population aged 65 or more is expected to increase by about 10% by 2070, it is important to think now about how this at-risk population can be assisted to drive safely, especially when performing accident-prone maneuvers, such as approaching and crossing an intersection. Studies have highlighted the benefits of driver assistance devices for senior drivers, undeniably improving safety and reducing the stress associated with driving (Schall et al., 2010).

In this context, we wished to carry out a reflection on the design of driving assistance systems (ADAS) adapted to the specificities and needs of older drivers when intercepting a moving inter-vehicular space. Although the experimental task used in the present study is relatively far from real driving tasks, the ability to intercept moving inter-vehicular gaps required in our study is paramount to successfully completing a variety of driving tasks. The main function of these ADAS is to compensate for the diminished perceptual-motor abilities of older drivers, in order to secure the crossing of an intersection (Vrkljan and Miller-Polgar, 2005). It has been shown that when ADAS are designed and used appropriately, they have the potential to help drivers cope with the complex demands of driving (Vrkljan and Miller-Polgar, 2005; Young et al., 2016). An ADAS designed following a user-centered approach could (i) reduce the occurrence of accidents by compensating for age-related perceptual-motor declines (Caird, 2004; Davidse et al., 2009; François et al., 2016) and consequently (ii) delay the cessation of driving, which is synonymous with loss of autonomy and sociability (Koppel et al., 2009; Koppel and Charlton, 2013). Following a user-centered approach, the ADAS design process was initiated by the characterization of older drivers’ behaviors when approaching and crossing an intersection.

The driving simulator study conducted by Tran Van et al. (2022) aimed to analyze travel speed regulations implemented by older drivers when intercepting a moving inter-vehicular space. The task constraints were manipulated by varying the initial distance between the participants and the interval to be intercepted. The behavior of older drivers was compared with that of younger drivers. Analysis revealed, under most experimental conditions that were tested, gradual and systematic early speed adjustments by older drivers, comparable to that produced by younger drivers in previous studies (e.g., Louveton et al., 2012a,b; Mathieu et al., 2017a,b). These speed adjustments are functional because they allow the driver to intercept the inter vehicular interval at a specific location (i.e., near the middle of the interval) by gradually reducing the experimentally induced offset during travel. From one trial to another, different offsets give rise to different displacement speed adjustments so as to cross the interval near the same location. As a consequence, a concomitant analysis of the patterns of between-trial variability of the travel speed and the current deviation (i.e., the location of the crossing point of the interval if the travel speed has not changed) has revealed opposing patterns of change over time (Bootsma and Van Wieringen, 1990; Bardy and Laurent, 1998; Chardenon et al., 2002; Louveton et al., 2012a,b; Mathieu et al., 2017a,b). Successive speed adjustments (increase in displacement speed variability) make it possible to gradually reduce the gap relative to the targeted crossing point (decrease in current deviation variability). These results argue for the implementation of a functional coupling between perception and action (Gibson, 1979; Michaels and Carello, 1981; Warren, 2006). Information present in the optical flow appears to inform the agent about the nature of the regulations to be produced (in the task of interest: accelerate, decelerate or maintain the speed of travel unchanged) so that the control of the speed of travel results from the detection of this information (Gibson, 1979; Warren, 1988, 2006; Bootsma, 1998). The information used to control the speed of travel when intercepting the interval could be the rate of change of the angle subtended by the driver’s eye between the direction of travel and, for example, the center of the inter-vehicular space (i.e., bearing angle) (Lenoir et al., 1999a,b; Chardenon et al., 2002). Control of intersection crossing would depend on the ability of drivers to regulate their travel speed according to the rate of change of the bearing angle, and a close coupling between perception and action would allow this task to be accomplished. Tran Van et al. (2022) identified difficulties for older drivers when a large increase in speed was required to successfully complete the task and showed that in these experimental conditions, the rate of change of the bearing angle is the lowest and probably the most difficult to perceive for older drivers. These difficulties interfere with the implementation of the perception-action cycle and lead older drivers to initiate their regulations later and to produce fewer safe behaviors relative to younger drivers.

Thus, an ADAS that would allow older drivers to know at any time whether to accelerate, decelerate or maintain speed to complete the task could be very useful. This augmented information, redundant with the information about the rate of change of the bearing angle, would make it possible to preserve the functional relations between perception and action, even when the rate of change of the bearing angle is difficult to discriminate.

The literature about the development of ADAS that can assist drivers during intersection crossing is growing (e.g., Brown et al., 2005; Chen et al., 2011; Bao et al., 2012; Houtenbos et al., 2017). However, few studies have aimed to design an ADAS that acts as an enhancement of the perception of the environment, labelled ‘perceptual mode’ in the human-machine cooperation literature (Hoc et al., 2009; Navarro et al., 2011). A notable exception is the study by Dotzauer et al. (2013) which tested the effect of an ADAS on elderly people to assist them in crossing an intersection while visibility was poor, forcing the driver to slow down before crossing. The ADAS indicated whether the size of the gap to the crossing traffic was sufficient to safely cross the intersection. The concurrent feedback provided by the ADAS was a gauge located on the dashboard whose colour gave an indication about the dangerousness of the crossing maneuver. Behavioral changes were reported in the presence of the ADAS but they did not allow the authors to conclude whether these changes corresponded to a safer and more efficient way of driving or whether they reflected risky behavior induced by ADAS use. However, they reveal that an ADAS can influence behavior and performance in positive or negative ways, making pre-design thinking important.

Some studies on goal-directed locomotor tasks have tested on-line feedback (i.e., concurrent feedbacks) to enhance perception of the environment (Janelle et al., 1997; Chiviacowsky and Wulf, 2002, 2005; Camachon et al., 2007; Huet et al., 2009). In Huet et al.’s (2009) study, participants received concurrent feedback about the error that would be observed in a goal-directed locomotion task, if the current walking speed remained constant. This means that participants were continuously informed of the discrepancy between the current behavior and the behavior required to achieve the desired goal. The results reveal the positive effect of concurrent feedback on performance and consequently on the implementation of the perception-action cycle.

Returning now to our baseline task (i.e., intercepting a moving space while driving), our aim here is to test the effectiveness and acceptance of a driving assistance system in the form of a concurrent feedback during task completion that should enable older drivers to perform the task. The participants received concurrent feedback about the current deviation, i.e., about the need to increase their speed, decrease it or keep it unchanged, in order to succeed in the task. We decided to implement this feedback in two different ways. Another underlying dimension of our study was to compare how display form impacts performance. If we had adopted a single display style and the results had been negative, it would have been complicated to determine the origin of this drop in performance, i.e., whether it was due to the information provided or to the type of display used. The first ADAS (Head Down)** tested was in the form of a gauge located on the dashboard. Its use required an alternation of visual anchoring on the flow of incoming traffic and on the dashboard. The second ADAS (Head Up)** was embedded in the oncoming traffic, so as to provide the driver with all the information he/she needed to adjust their speed on the driving scene. Our study aimed not only to test the effectiveness of the two ADAS but also to measure their acceptability by drivers. We considered this last measure important because it is recognised as a prerequisite for the successful introduction of ADAS, and its evaluation allows us to estimate drivers’ willingness to use these systems (Najm et al., 2006). The most widely used model and the one we used in this study to investigate technology acceptance, is the technology acceptance model (TAM) (Davis, 1989; Manis and Choi, 2019).

By implementing the ADAS, our intention was to help older drivers to perform the crossing task. Both ADAS were designed to facilitate the implementation of the necessary speed regulations to cross the intersection safely. The Head Down was expected to attract drivers’ glances, and thus intermittently divert them from the visual information available in the driving scene. Such visual diversion was not hypothesised for the Head Up, which should allow drivers to benefit from the perceptual aid provided by the ADAS while maintaining their gaze on the flow of incoming traffic.

Because the ADAS were designed as perceptual mode assistance systems aiming to enhance drivers’ capabilities, good levels of acceptance were hypothesised, higher for older drivers than for younger drivers, which is consistent with recent studies on acceptance of automotive technology (Son et al., 2015; Lee et al., 2017).

## 2. Materials and methods

### 2.1. Participants

Fourteen young drivers (26 years ±3 years) and fourteen senior drivers (73 years ±5 years) participated in this study. Participants were required to have normal or corrected-to-normal vision, a minimum of 2 years of driving experience, and to drive a minimum of 20 kilometers per week. Seniors had to score above 24 on the Mini-Mental-State Examination (MMSE) to be retained. The study was conducted in accordance with the ethical guidelines of the Ethics Committee for Research in Science and Technology of Physical and Sports Activities (CERSTAPS: IRB00012476-2022-20-03-168) and in compliance with the Declaration of Helsinki for human research and the international principles governing research on humans.

### 2.2. Apparatus

A fixed-base driving simulator was used in this study. The position of the seat and pedals were adjustable, allowing the driving interface settings to be tailored to the size of the participant. An automatic gearbox was used, so only accelerator and brake pedals were available.

This driving device combined a set of pedals (Extreme Competition Control, Minneapolis, United States) and a steering wheel (ECCI’s Trackstar 6,000) with a virtual reality application developed in-house (ICE© software) running on a PC (Microsoft Windows 10 Pro, Intel Core i9¬9,900 processors (8 curs, 3.1–5.0 Ghz Turbo, 16 MB cache, NVIDIA Geforce RTX2080 Super graphics card)). The in-house ICE© software generated the virtual environment. It was used to create virtual reality environments combining visual and auditory content, program the scenarios, synchronize the driving interface and the virtual reality headset, and perform data acquisition (Coutton-Jean et al., 2009; Marti et al., 2014).

A virtual reality headset (HTC Vive® Pro Eye) projected the virtual environment with a screen resolution of 2,880 × 1,600 pixels and 615 PPI and an eye-tracking system (optimised by Tobii®). The headset offered a field of view of 110 degrees. The eye-tracking was done on these 110 degrees and offered a precision of 0.5° to 1.1°. This allowed the recording of the coordinates of the gaze direction at a sampling frequency of 120 Hz.

### 2.3. Visual environment

The rural environment in which the participants were travelling consisted of a section of straight lane of a two-lane road separated by a solid white line and bounded by a broken bank line. A right-angle intersection with another two-lane road at a variable distance was programmed. A train of vehicles could approach the intersection from the left.

### 2.4. Experimental design

**Figure 1:**
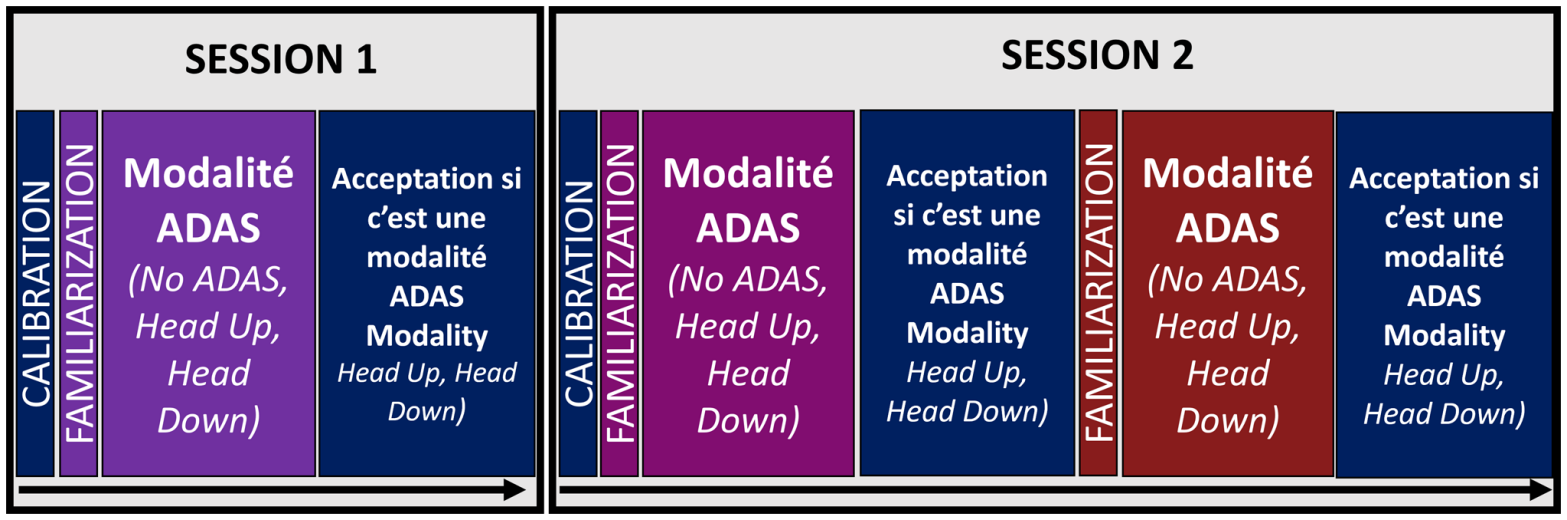
Chronological course of the experiment. Session 1 lasted 2 h and then a break of 24 h minimum was left before carrying out session 2 which also lasted 2 h.

The experiment was divided into two sessions of 2 hours separated by 24 h. Each session began with a task designed to familiarize the drivers with the driving simulation before the actual experiment. This calibration phase was designed to help drivers familiarize themselves with the acceleration and deceleration capabilities of the vehicle they would be using for the remainder of the experiment (Figure 1). Once the calibration task had been successfully completed to calibrate with the device, testing of the driver assistance systems was ready to begin. Each ADAS condition was divided into a familiarization phase, an experimental phase and a phase in which acceptance was measured (for the Head Down and Head Up conditions). In the first session, one ADAS condition was run on top of the calibration task, while the second session contained the other two ADAS conditions. The order of the ADAS conditions (i.e., No-ADAS, Head Down, Head Up) was randomized.

### 2.5. Calibration phase

#### 2.5.1. Task

In this phase, participants were asked to drive on a straight road and to maintain a constant distance behind a car moving in front of them changing speed (see Mathieu et al., 2017a,b; Tran Van et al., 2022).

#### 2.5.2. Procedure

The calibration was composed of 3 sessions of 2 min each. The participant received concurrent feedback during the first minute. This feedback takes the form of a vertical gauge located slightly on the left of the steering wheel. This gauge contained a cursor which moved along the gauge and indicated the current inter-vehicular distance. This information indicated the nature of the regulation to be produced (moving closer to the vehicle, moving away or maintaining the same position). This feedback disappeared during the second minute of the trial.

The participant was ‘calibrated’ when he/she obtained in at least 2 of 3 trials a percentage of time during which the prescribed distance was maintained without feedback (i.e., during the second minute of the trial) greater than 80% (Tran Van et al., 2022). After the calibration task, the participant had 5 min of rest before starting the experimental phase.

### 2.6. Experimental phase (moving gap interception)

#### 2.6.1. Task

**Figure 2:**
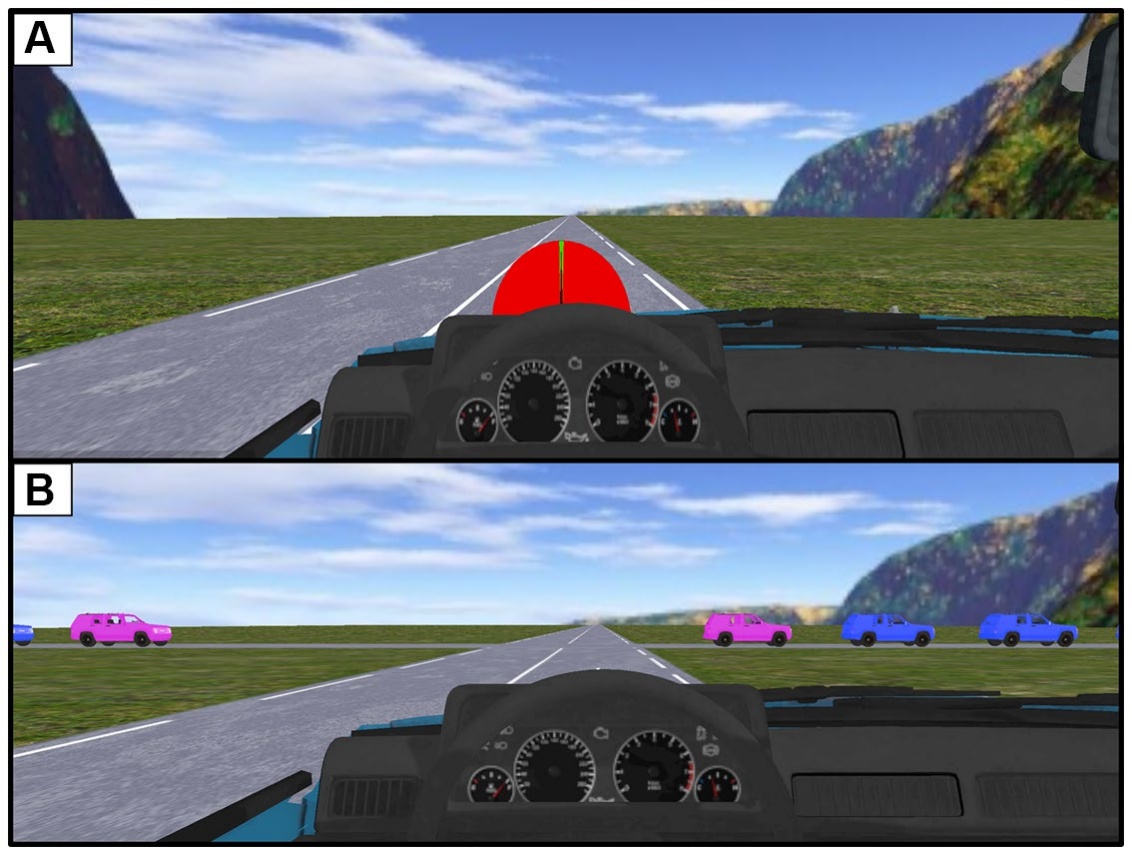
Illustration of the virtual environment in which the driver moves. **(A)** Location and shape of the two-coloured speedometer displayed during the first part of the task. **(B)** Representation of the train of vehicles approaching the intersection and materialization of the inter-vehicular space to be crossed (between the two purple cars).

The task used here was similar to that used in the studies by Tran Van et al. (2022), Mathieu et al. (2017a,b), and Louveton et al. (2012a,b, 2018). Participants were asked to drive through an intersection that was being approached by a train of vehicles on the left. They had to pass through the space between the two purple vehicles within the vehicle train. A total of 6 SUVs (length: 4.205 m, width: 1.80 m, height: 1.70 m) made up the vehicle train, which was moving at a speed of 10 m/s (36 km/h). The interval to be crossed was 27 meters (2.7 s) (Figure 2B).

#### 2.6.2. Procedure

At the beginning of each trial, the participant was asked to start and accelerate to a stabilized speed of 16 m/s. The driver had concurrent feedback in the form of a two-coloured speedometer to inform him about the difference between his current speed and the target speed (Figure 1A). The position of the needle on the speedometer informed him about the type of regulation to be made to reach the prescribed speed. When the arrow was held in the target (green) area for at least 5 s the speedometer disappeared, and the crossing scenario began. The initial conditions of the task (i.e., participants’ initial speed) were standardized for each experimental modality through this procedure. In the case the driver collided with a vehicle an audible warning sounded.

### 2.7. Independent variable

#### 2.7.1. Offset

An offset between the participant’s expected arrival time at the intersection (if the initial speed was held constant) and the arrival time of the center of the interval to be crossed was created by manipulating the initial distance between the participants and the intersection. An initial distance (150 m) was assigned for the ‘no offset’ condition (Offset 0 s) so that the participant could cross the intersection at the center of the inter-vehicular space while keeping his initial speed (i.e., 16 m/s or 57.6 km/h) unchanged. Distances (118, 134, 166 and 182 m) were attributed corresponding, respectively, to the 4 other conditions (Offset −2, −1, 1 and 2 s) in order to require distinct speed regulations. To cross the intersection safely the participant had to decelerate or accelerate when confronted with a positive or negative offset.

Advanced Driver Assistance Systems (ADAS).

Three ADAS were tested, namely No-ADAS, Head Down and Head Up.

No-ADAS. The first modality served as a control condition in which participants performed the task without an ADAS.

In the other two modalities, two specific ADAS were developed and tested to provide concurrent feedback to the participants during the task completion. This concurrent feedback represents the driver’s crossing position at each instant in time if the current displacement speed remained constant. This allowed the participant to know the type of regulation to be made but also the amount of regulation needed. The two ADAS differed in the way this augmented information was made available.

#### 2.7.2. Head Down

**Figure 3:**
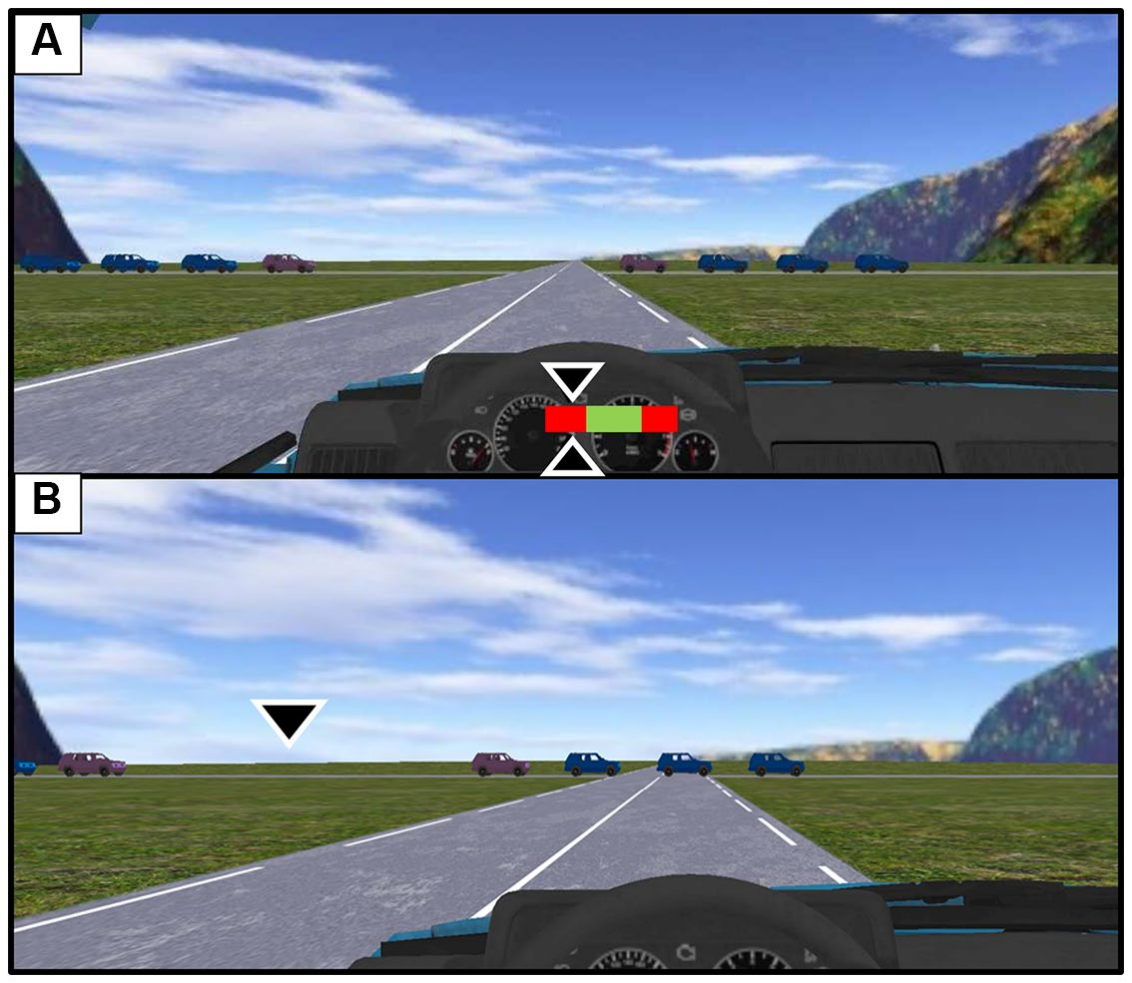
Illustration of the concurrent feedback provided to drivers. **(A)** Concurrent feedback provided by the Head Down. In this situation, the driver needs to accelerate in order to cross the intersection without colliding with the vehicle that closes the inter-vehicular gap. **(B)** Concurrent feedback provided by Head Up. In this situation, if the driver keeps its current speed unchanged the driver will cross the interval at the location indicated by the cursor. (N.B: The size of the arrows is amplified in comparison with the experiment to increase visibility).

The ADAS consisted of a two-coloured horizontal gauge on which a cursor moved (Figure 3). The gauge can be seen as an abstraction of the traffic flow, with the green part representing the inter-vehicular gap to be crossed and the two red parts the vehicles delimiting the interval to be crossed. The moving cursor indicates at each instant the gap crossing location if the current displacement speed remained constant. Of course, each time the displacement speed changes, the location of the cursor changes accordingly. As a consequence, when the cursor is located in the left red part of the gauge, it indicated that if there were no acceleration, the participant would collide with the last part of the vehicle train (Figure 3A). The exact position of the cursor on the red area indicates the amount of acceleration required to succeed in the task. Conversely, if the cursor is in the right-hand red area of the gauge, it indicates that if there were no deceleration, the participant would collide with the first part of the vehicle train. According to the same logic, when the cursor is located in the green part of the gauge, the driver knows what would be the exact gap crossing location if the current speed were kept constant.

#### 2.7.3. Head Up

The specificity of this second design lies in the fact that the concurrent feedback related to the future gap crossing position is depicted in the traffic flow. This feedback is materialized by means of a cursor (Figure 3B). In the same way as for the previous ADAS, the location of the cursor informs the driver about the nature and the amplitude of the regulations to be produced, in order to cross the interval at a particular place (see Figure 3B).

In summary, both initial distances (Offsets −2, −1, 0, 1, 2 s) and ADAS modalities (No-ADAS, Head Down and Head Up) were manipulated. The participants (young and older drivers) performed 5 trials per condition for a total of 75 trials. The 75 trials were randomly distributed in 3 blocks of 25 trials, while the order of the blocks was counterbalanced.

### 2.8. Dependent variables

#### 2.8.1. Driving behavior

An analysis of participants’ behavior was performed through three indicators: gap crossing position, displacement speed and current deviation.

##### 2.8.1.1. Gap crossing position

The most macroscopic variable corresponds to the participants’ position in the inter-vehicular interval as they crossed the intersection. This position was determined for each trial of each modality to calculate the constant error (Schmidt and Lee, 2005). The constant error (CE) was an indicator of the performance produced that takes into account the sign of the errors. Taking the center of the inter-vehicular space as the origin, a negative crossing position revealed that participants crossed the intersection after the center of the inter-vehicular space (i.e., closer to the vehicle closing the gap). Similarly, a positive crossing position meant that participants crossed the intersection before the center of the inter-vehicular space (i.e., closer to the vehicle opening the gap).

##### 2.8.1.2. Displacement speed and displacement speed variability

The evolution of speed over time was analyzed to explore speed adjustments before intersection crossing. Each trial was synchronized by taking as a common reference (t0) the moment of crossing the gap. Then the speed profiles were discretized backwards for each trial starting from the intersection crossing time to obtain 7 time steps (i.e., 7–6 s, 6–5 s, 5–4 s, 4–3 s, 3–2 s, 2–1 s, 1–0 s). Then for each participant and for each time step an average speed and the inter-trial variability of the speed were calculated.

##### 2.8.1.3. Current deviation and current deviation variability

The current deviation represents at each time step the participant’s crossing position in the inter-vehicular space if the current speed were kept constant (Louveton et al., 2012a,b). This variable is calculated at each moment and fluctuates as the participants’ speed changes, indicating to what extent the speed changes produced are functional. In the ‘no offset’ condition the current deviation is zero at the beginning of the trial. Thus, if the initial speed were held constant throughout the trial, the participant would cross the intersection at the center of the inter-vehicular space. In the conditions with positive or negative offsets (i.e., −2, −1, 1, and 2 s) the initial current deviation was −2, −1, 1, and 2 s respectively, at the beginning of the test. Therefore, speed regulations were required to cross the intersection near the center of the inter-vehicular space. The objective of the analysis was precisely to describe the dynamics of the changes in current deviation. As for the speed profiles, after calculating the current deviation profiles, all trials were synchronized by taking as common base (t0) the time of crossing the interval. Then the current deviation profiles were discretized backwards from the time of intersection crossing to obtain 7 time steps (i.e., 7–6 s, 6–5 s, 5–4 s, 4–3 s, 3–2 s, 2–1 s, 1–0 s). Then for each participant and for each time step a mean current deviation as well as the inter-trial variability of the current deviation was calculated.

#### 2.8.2. Ocular behavior

**Figure 4:**
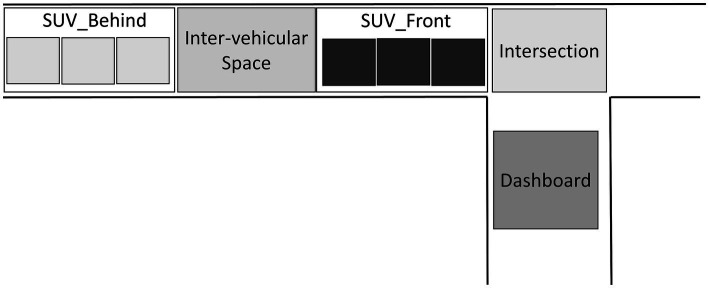
Schematic representation of the five areas of interest (AOIs) used in the experiment as part of the eye-tracking analysis: SUV_Behind: Last part of the train of vehicles; Inter-vehicular space; SUV_Front: First part of the train of vehicles; Intersection; Dashboard.

Drivers’ visual explorations were also analyzed. For this purpose, five areas of interest (AOIs) corresponding to the vehicle train located behind (SUV_Behind) or in front (SUV_Front) of the interval, to the inter-vehicular interval (Inter-vehicular space), to the junction of the two roads (Intersection) and on the dashboard (Dashboard) were defined (Figure 4). The gaze direction data collection was performed throughout the trial from the appearance of the intersection to the inter-vehicular gap crossing. From these data the average percentage of time spent in each AOI during a trial was determined (Navarro et al., 2019).

#### 2.8.3. Acceptance

Acceptance was measured after the use of each ADAS using a self-reported questionnaire to quantify the acceptance of both Head Down and Head Up displays after being used by the drivers, following the procedure of Huygelier et al. (2019).

The acceptance of ADAS was assessed through three variables: (1) Perceived Usefulness (PU), (2) Perceived Ease of Use (PEU) and (3) Intention to Use (IU) (Davis, 1989; Manis and Choi, 2019). Three items per variable were used. Participants were asked to respond to these items on a Likert scale from 1 (‘strongly disagree’) to 5 (‘strongly agree’). There follows an example of an item used for each of the three variables: the PU (‘I think this ADAS is useful for crossing intersections safely’), for the PEU (‘I think the operation of this ADAS is easily understood by me’), and for the IU (‘If I had the opportunity to have easy access to this ADAS, I would want to use it’). Internal consistency was good for each of three variables with Cronbach’s alpha ranging from 0.764 to 0.991 (Table 1).

**Table 1:** Cronbach’s alpha, mean and standard deviation (SD) of the three acceptance variables for younger and older drivers and for the ADAS tested: Perceived Usefulness (PU), Perceived Ease of Use (PEU) and Intention to Use (IU).

	Younger drivers				Older drivers			
	Head Down		Head Up		Head Down		Head Up	
	Cronbach’s alpha	Mean (SD)	Cronbach’s alpha	Mean (SD)	Cronbach’s alpha	Mean (SD)	Cronbach’s alpha	Mean (SD)
PU	0.954	2.97 (±1.52)	0.840	4.07 (±0.94)	0.966	4.11 (±1.32)	0.935	4.45 (±0.88)
PEU	0.895	4.14 (±1.22)	0.764	4.83 (±0.37)	0.987	4.57 (±1.24)	0.896	4.78 (±0.41)
IU	0.971	2.21 (±1.22)	0.943	2.85 (±1.22)	0.996	4.04 (±1.34)	0.991	4.09 (±1.3)

### 2.9. Statistical analyses

Statistical analyses were performed using repeated measures ANOVA. The Shapiro–wilk test was used for each dependent variable to assess normality. In light of the observation that the data related to speed, variability of current deviation, and the percentage of time spent in each area of interest do not follow to a normal distribution, we used the Greenhouse–Geisser correction. For the gap crossing position, Population (Younger, Older) was used as a between subject factor, and both Offset (−2, −1, 0, 1, 2) and ADAS condition (No ADAS, Head Down, Head Up) as a within subject factor. For the time course of both speed and current deviation and their variabilities, Population (Younger, Older) was used as a between subject factor and Offset (−2,-1,0,1,2), Time (from 7 s from gap interception to interception in intervals of 1 s gap crossing, i.e., 7 bins) and ADAS condition (No ADAS, Head Down, Head Up) as within subject factors. For the average percentage of time spent in each AOI, Population (Younger, Older) was used as a between subject factor and Offset (−2,-1,0,1,2), AOI and ADAS condition (No ADAS, Head Down, Head Up) as within subject factors. In case the results were significant, post-hoc analyses were performed using Holm’s test. For psychological variables, one-sample t-test were performed on each variable to see of the scores were significantly different from the scale mean. Paired sample t-tests were then performed to identify differences in ADAS conditions within a population.

## 3. Results

### 3.1. Success rate

During the experiment, the intersection was crossed 490 times by the older drivers and 490 times by the younger drivers. There were no crashes for the younger drivers while there were 6 crashes for the older drivers (98.78% success rate). These trials were removed from further analysis.

### 3.2. Gap crossing position

The analysis of variance on gap crossing position revealed a significant main effect of Offset (*F* (4,104) = 69.997, *p* < 0.001, ꞃ2 = 0.316) and ADAS (*F* (2,52) = 9.185, *p* < 0.001, ꞃ2 = 0.040). Furthermore, the first-order interaction Offset*ADAS (*F* (8,208) = 2.141, *p* < 0.001, ꞃ2 = 0.014) was significant.

**Figure 5:**
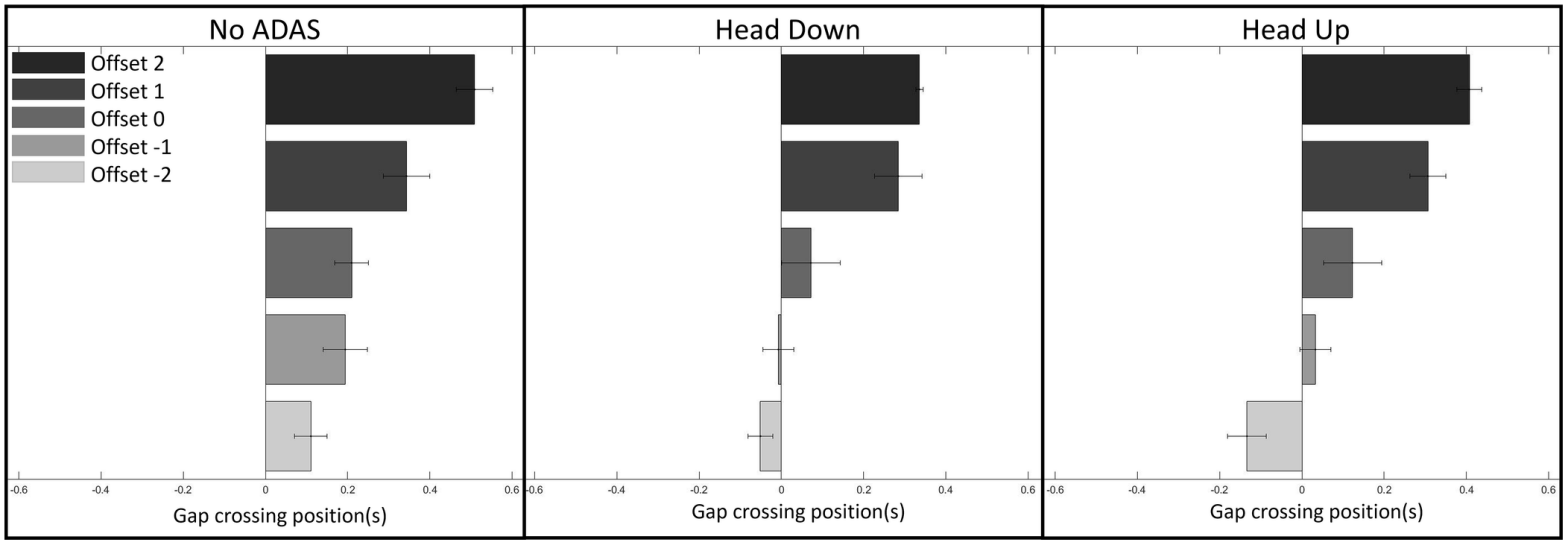
Gap Crossing Position (constant error) as a function of the Offset and ADAS.

Post-hoc comparisons on the Offset*ADAS interaction did not reveal any significant differences. However, post-hoc comparisons on ADAS revealed that gap crossing positions were different for Head Down (5.4 m; 0.15 s) and Head Up (7.2 m; 0.2 s) conditions in comparison with No-ADAS condition (9.8 m; 0.27 s) (Figure 5). Finally, post-hoc comparisons on Offset revealed that the gap crossing position differed for each Offset condition (all *p* < 0.0001). Gap crossing position converged progressively towards the center of the interval, ranging from 0.4 s for the Offset 2 condition to −0.08 s for the Offset −2 condition.

### 3.3. Speed profiles

**Figure 6:**
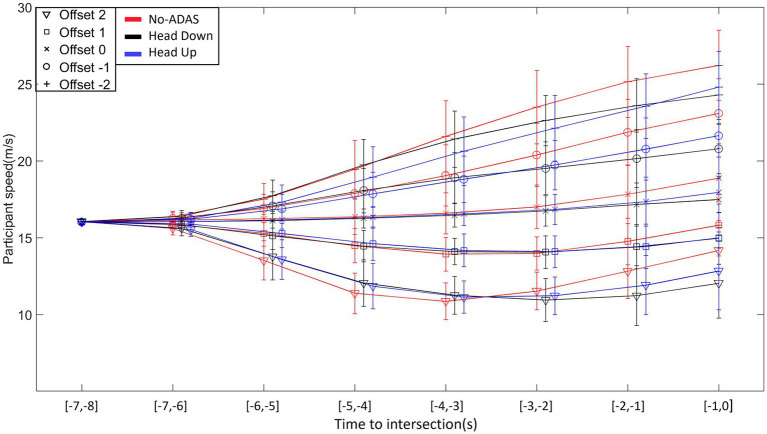
Time course of the participants’ average speed in the different Offset and ADAS conditions.

As can be seen in Figure 6, Offset influences the evolution of displacement speed over time and gives rise to specific profiles. Participants modulate their displacement speed very early on, in accordance with task constraints. Two seconds after the appearance of the train vehicle, speed profiles begin to differentiate. Positive Offsets give rise to a decrease in displacement speed, more pronounced as the Offset rises. The opposite is true for negative Offsets with an increase in displacement speed more pronounced as the Offset rises.

The analysis of variance on displacement speed confirmed these results and revealed a significant main effect of Offset (*F* (4, 104) = 508.332, *p* < 0.001, ꞃ2 = 0.188), Time (*F* (7,182) = 66.194, p < 0.001, ꞃ2 = 0.086) and ADAS (*F* (2, 52) = 14.901, *p* < 0.001, ꞃ2 = 0.003). The second-order interactions Offset*Time*ADAS (*F* (56, 1,456) = 5.431, *p* < 0.001, ꞃ2 = 0.002), Offset*Time*Population (*F* (28, 728) = 2.688, *p* < 0.001, ꞃ2 = 0.002) and Offset*ADAS*Population (*F* (8, 208) = 2.326, *p* < 0.021, ꞃ2 = 4.199 x^10–4) were all significant.

Post-hoc comparisons on the Offset*Time*ADAS interaction indicate a more pronounced increase in speed for Offset −2 during the last 3 s before crossing in the No-ADAS condition in comparison with both Head Down and Head Up conditions. A similar behavior is observed for Offset −1 from 2 s before crossing onwards. The decomposition of the other interactions did not reveal significant effects.

### 3.4. Current deviation profiles

**Figure 7:**
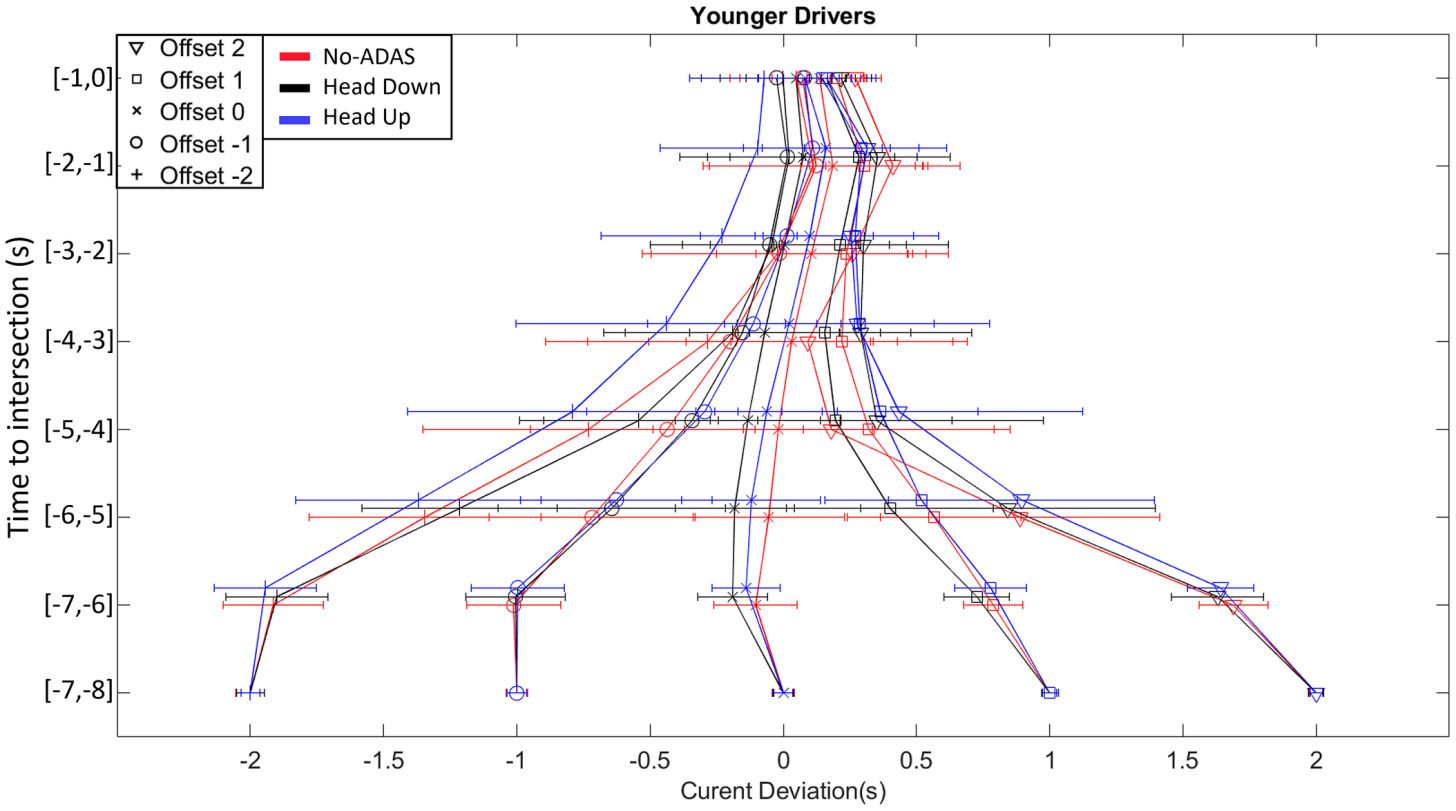
Time course of the current deviation for each Offset condition (from Offset −2 to Offset 2), for each ADAS (No-ADAS, Head Down, Head Up) for Younger drivers.

**Figure 8:**
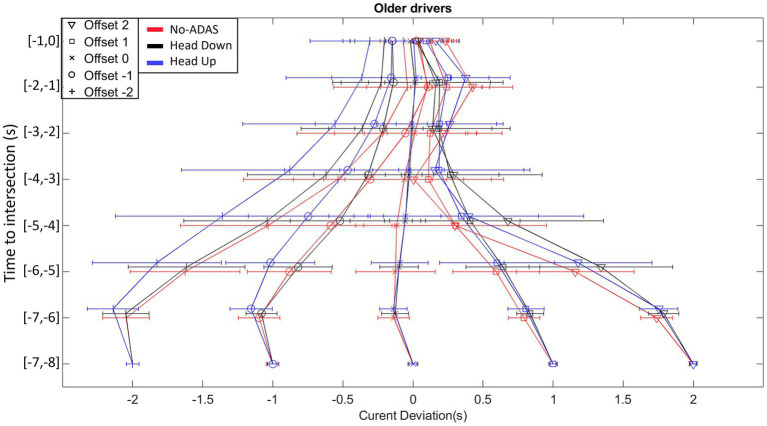
Time course of the current deviation for each Offset condition (from Offset −2 to Offset 2), for each ADAS (No-ADAS, Head Down, Head Up) for Older drivers.

As can be seen in Figures 7, 8, the current deviation profiles exhibit a gradual convergence towards the chosen gap crossing location. Offset manipulations allowed us to place the participants in different situations requiring the production of distinct speed regulations. The convergence in current deviations whatever the Offset condition provides evidence that the speed changes described in the previous section are functional because they result in a reduction of current deviation.

The analysis of variance on the current deviation reveals significant main effects of Population (*F* (1, 26) = 4.931, *p* < 0.035, ꞃ2 = 0.004), Offset (*F* (4, 104) = 572.534, *p* < 0.001, ꞃ2 = 0.515) and Time (*F* (7, 182) = 31.981, *p* < 0.001, ꞃ2 = 0.022). The second-order interactions Offset*ADAS*Population (*F* (8, 208) = 2.778, *p* < 0.006, ꞃ2 = 0.001), Offset*ADAS*Time (*F* (56, 1,456) = 2.476, p < 0.001, ꞃ2 = 8.253×10^-4) and Offset*Time*Population (*F* (28, 728) = 3.615, *p* < 0.001, ꞃ2 = 0.003) were all significant.

Post-hoc comparisons on Offset*ADAS*Time and Offset*Time*Population did not reveal significant differences. However, post-hoc comparisons on Offset*ADAS*Population indicated that for Offset −2, the mean current deviation differs between No-ADAS and the other two conditions (Head Down and Head Up) in older drivers. The mean current deviation is smaller with the Head Down (−1.06 s) and Head Up (0.92 s) conditions in comparison with the No-ADAS (−0.80 s) condition.

### 3.5. Speed variability

The analysis of variance on inter-trial speed variability revealed significant main effects of Offset (*F* (4, 104) = 35. 262, *p* < 0.001, ꞃ2 = 0.050), Time (*F* (7, 182) = 216.637, p < 0.001, ꞃ2 = 0.352), and ADAS (*F* (2, 52) = 8.807, *p* < 0.001, ꞃ2 = 0.019). The first-order interactions Offset*Time (*F* (28, 728) = 14,933, *p* < 0.001, ꞃ2 = 0.040), Offset*ADAS (F (8, 208) = 2,277, *p* < 0.023, ꞃ2 = 0.006), and Time*ADAS (*F* (14, 364) = 5,017, *p* < 0.001, ꞃ2 = 0.010) were all significant.

**Figure 9:**
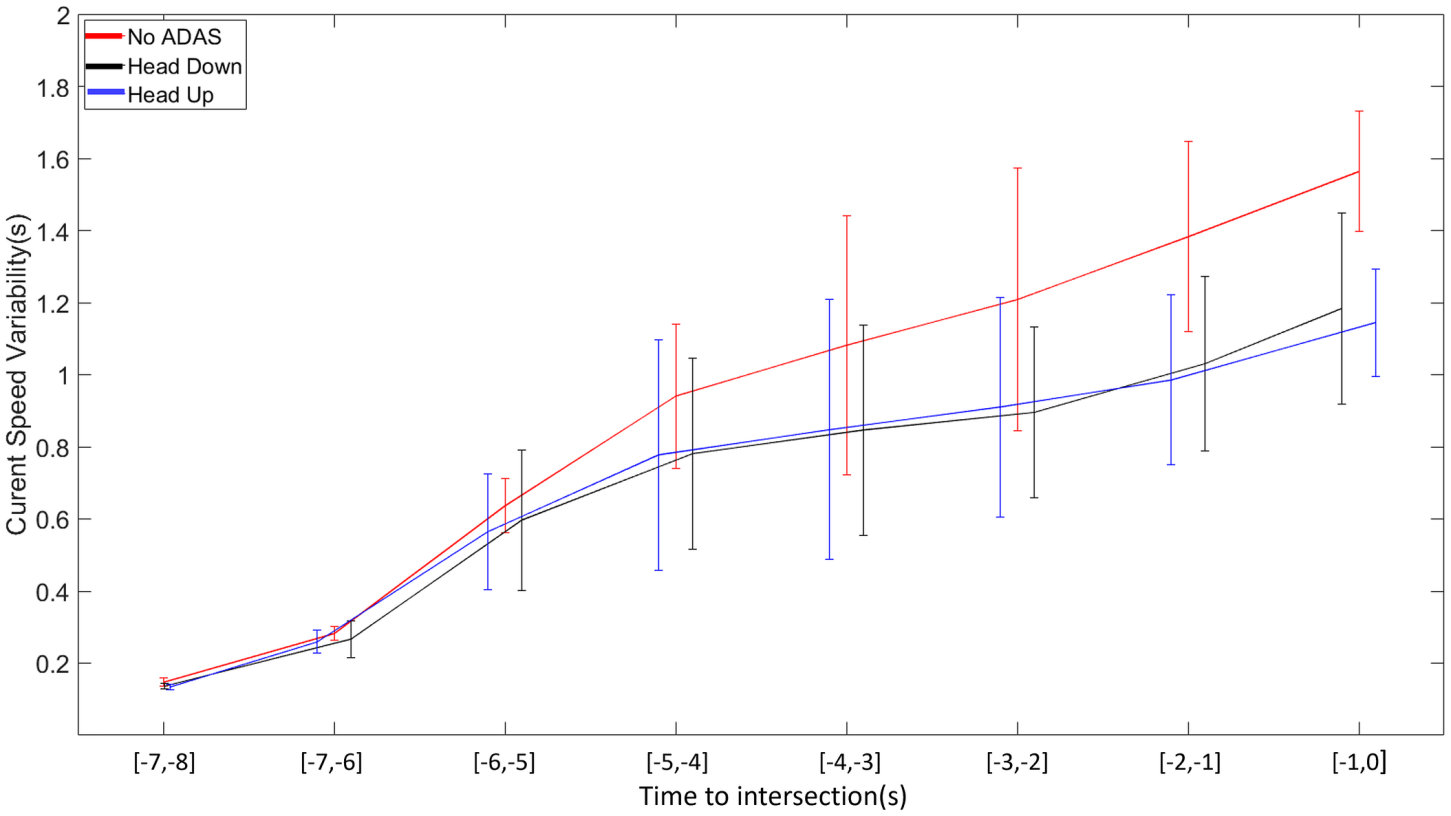
Average intra-participant speed variability as a function of time to intersection for each ADAS (No-ADAS, Head Down, Head Up).

As illustrated in Figure 9, displacement speed variability gradually increases over time whatever the ADAS condition. Post-hoc comparisons performed on the Time*ADAS interaction revealed a more pronounced increase in displacement speed in the No-ADAS condition from 3 s before the crossing onwards, in comparison with both Head Down and Head Up conditions. The decomposition of the other interactions did not reveal significant effects.

### 3.6. Current deviation variability

The analysis of variance on the inter-trial variability of current deviation revealed significant main effects of Offset (*F* (4, 104) =21.779, *p* < 0.001, ꞃ2 = 0.056), Time (*F* (7, 3,182) = 159.833, *p* < 0.001, ꞃ2 = 0.302), and ADAS (*F* (2, 52) = 4.666, *p* < 0.014, ꞃ2 = 0.009). The first-order interactions Offset*Time (*F* (28, 728) = 13.258, *p* < 0.001, ꞃ2 = 0.035) and Time*ADAS (F (14, 364) = 3.717, *p* < 0.001, ꞃ2 = 0.008) were all significant.

**Figure 10:**
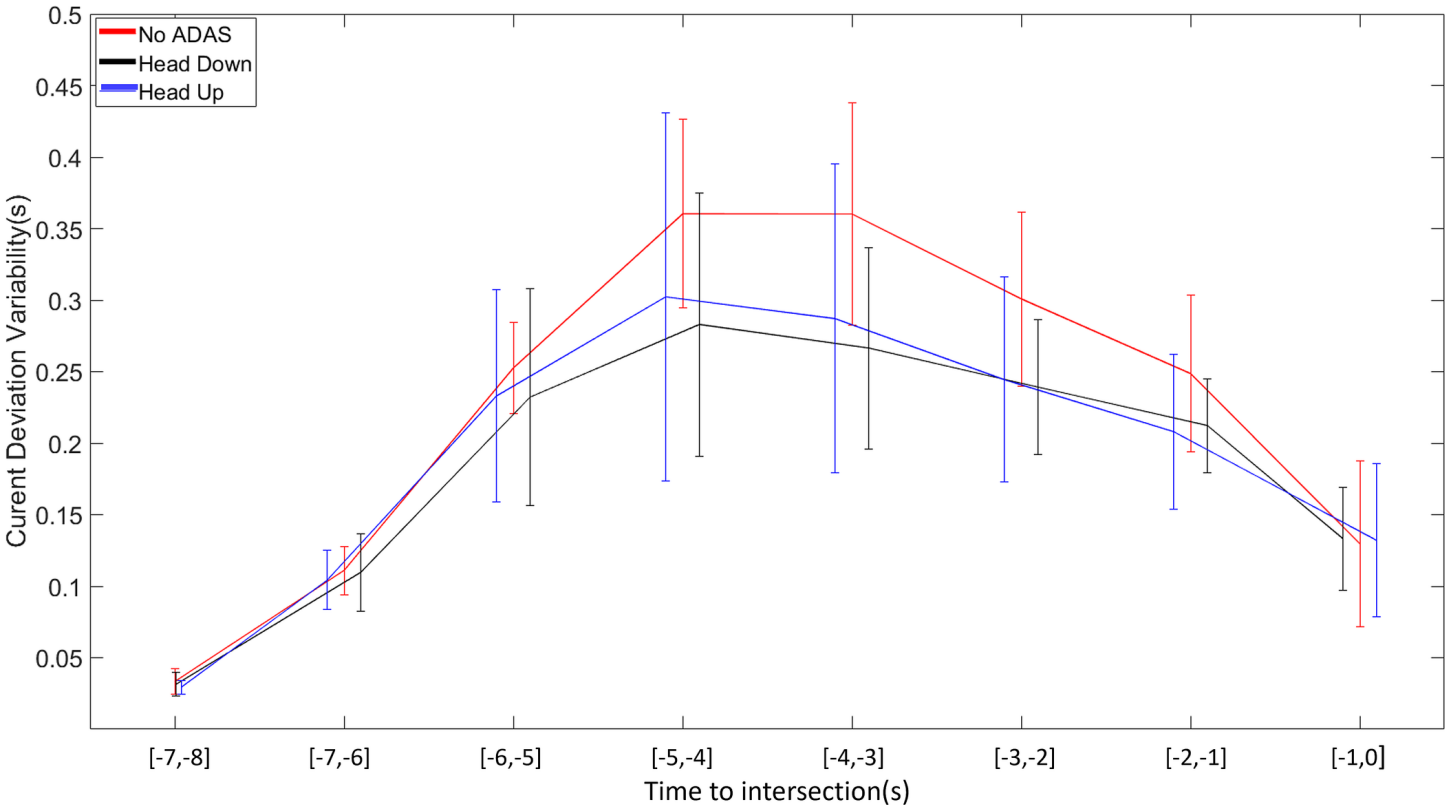
Average variability of intra-participant current deviation as a function of time to intersection for each ADAS (No-ADAS, Head Down, Head Up).

As can be seen in Figure 10, the variability of the current deviation exhibits a bell-shaped profile with a pronounced increase during the first part of the approach followed by a rather gradual decrease in the second part of the approach until the intersection is crossed. Post-hoc comparisons performed on Time*ADAS interaction reveal that 4 s before intersection crossing the current deviation variability is more pronounced in the No-ADAS condition in comparison with both Head Down and Head Up conditions.

### 3.7. Ocular behavior

Percentage of time spent within in each Area of Interest.

#### 3.7.1. No-ADAS

**Figure 11:**
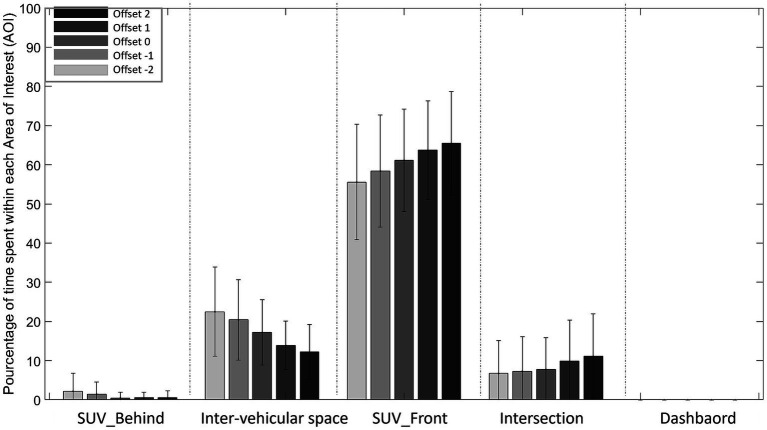
Percentage of time spent in each Area of Interest (AOI) for each Offset (from Offset −2 to Offset 2) in the No-ADAS condition. (NB: Dashboard is not viewed, so its value is 0%).

Regardless of condition, the vehicle train located in front (SUV_Front) is the area primarily viewed followed by inter-vehicular space, then intersection, then the vehicle train located behind (SUV_Behind) and dashboard (Figure 11).

The analysis of variance on the percentage of time spent in each area revealed significant main effects of AOI (*F* (4, 104) =312.923, *p* < 0.001, ꞃ2 = 0.886) and Offset (F (4, 104) =3.362, *p* < 0.012, ꞃ2 = 8.453e-5). The first-order Offset*AOI interaction (*F* (16, 416) = 19.300 p < 0.001, ꞃ2 = 0.013) was significant.

Post-hoc comparisons performed on Offset*AOI interaction revealed that participants spent less time fixating the vehicle train located in front (SUV_Front) with Offsets −2, −1 and 0 conditions in comparison with Offset 2 (*p* < 0.05). Moreover, participants spent less time fixating inter-vehicular space in Offsets 1 and 2 conditions in comparison with Offset −1 and − 2.

#### 3.7.2. Head Down

**Figure 12:**
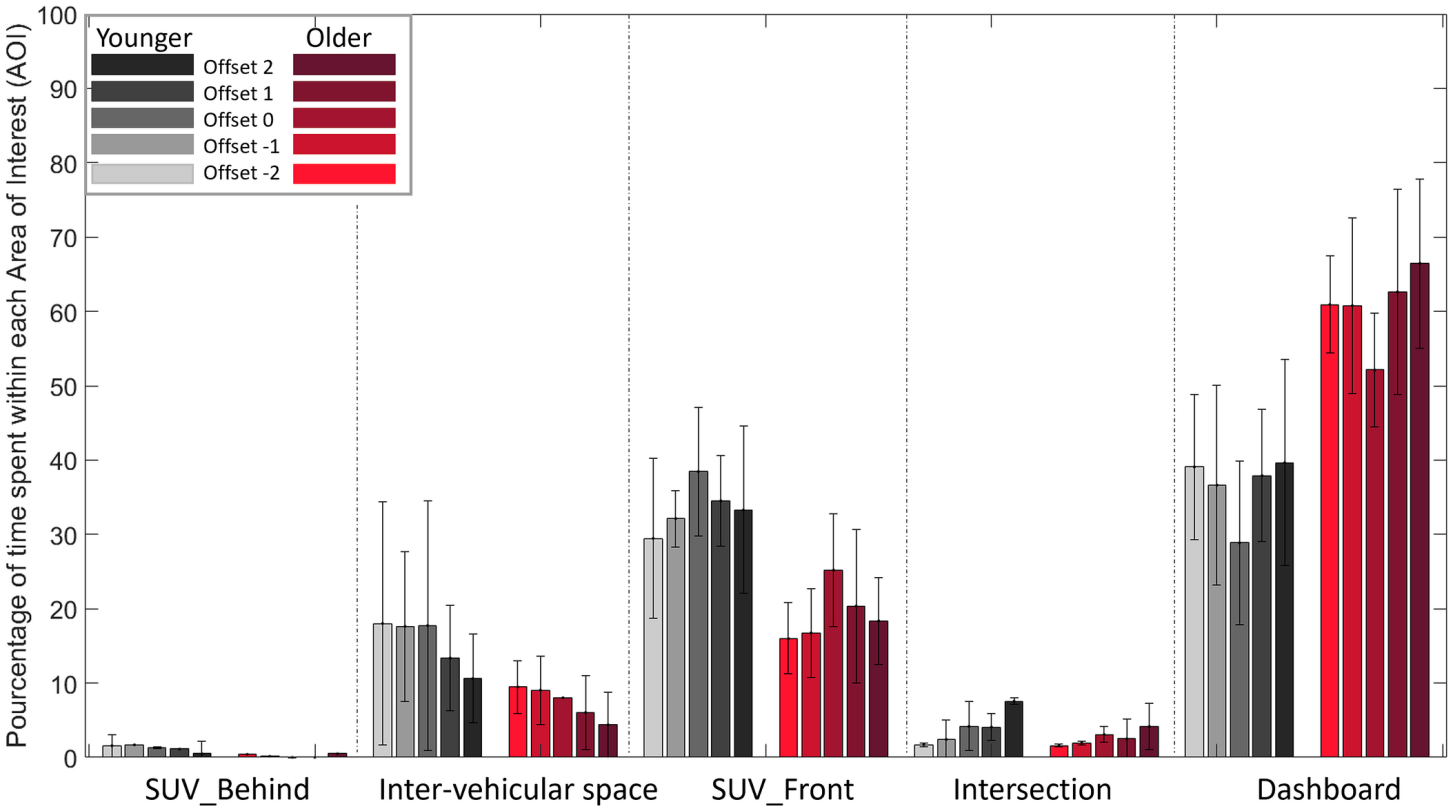
Percentage of time spent in each Area of Interest (AOI) for each Offset (from Offset −2 to Offset 2) in the Head Down condition.

As can be seen in Figure 12, regardless of condition and population, dashboard is the area primarily viewed followed by the vehicle train located in front (SUV_Front), then inter-vehicular space, then intersection and the vehicle train located behind (SUV_Behind).

The analysis of variance on the percentage of time spent in each zone revealed a significant main effect of AOI (*F* (4, 104) =42.316, *p* < 0.001, ꞃ2 = 0.544) and Offset (*F* (4, 104) =9.780, *p* < 0.001, ꞃ2 = 1.161e-4). The first-order interactions Offset*Population (*F* (4, 104) =3.194, *p* < 0.001, ꞃ2 = 3.791e-5), AOI*Population (*F* (4, 104) =5.789 *p* < 0.001, ꞃ2 = 0.074) and AOI*Offset (*F* (16, 416) =9.689, *p* < 0.001, ꞃ2 = 0.012) were all significant.

Post-hoc comparisons on the Offset*Population interaction did not reveal significant differences. In contrast, post-hoc comparisons on AOI*Population interaction revealed a significant difference between younger and older drivers in the percentage of time spent looking at the dashboard: older drivers (61.9%) spent more time looking at the dashboard than younger drivers (35.8%).

#### 3.7.3. Head Up

**Figure 13:**
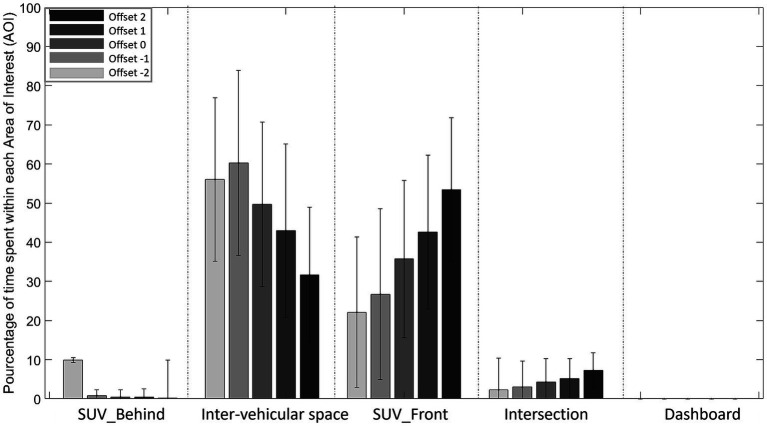
Percentage of time spent in each Area of Interest (AOI) for each Offset (from Offset −2 to Offset 2) in the Head Up condition. (NB: Dashboard Down is not viewed, so its value is 0%).

As can be seen in Figure 13, regardless of condition, inter-vehicular space is the area primarily viewed, followed by the vehicle train located in front (SUV_Front), then intersection, then the vehicle train located behind (SUV_Behind) and dashboard.

The analysis of variance on the percentage of time spent in each zone revealed significant main effects of AOI (*F* (4, 104) =81.615, *p* < 0.001, ꞃ2 = 0.629) and Offset (*F* (4, 104) = 2.645, *p* < 0.038, ꞃ2 = 7.075e-5). The first-order Offset*AOI interaction (*F* (16, 416) = 47.572 *p* < 0.001, ꞃ2 = 0.098) was significant.

Post-hoc comparisons performed on Offset*AOI interaction revealed that participants spent more time fixating inter-vehicular space in Offsets 0, −1 and − 2 in comparison with Offsets 2 and 1 (p < 0.05). In contrast, the opposite was observed for the vehicle train located in front (SUV_Front). Participants spent more time fixating the vehicle train located in front (SUV_Front) with Offsets 2 and 1 in comparison with Offsets 0, −1 and − 2.

### 3.8. Acceptance

#### 3.8.1. Younger drivers

Acceptance of ADAS by younger drivers is contrasted.

PU of Head Down was not different from the scale mean (*p* = 0.953), indicating that younger drivers found this ADAS neither useful nor useless (see Supplementary Table S1). PU of Head Up was significantly different from the scale mean (*p* = 0.001), indicating that younger drivers found the Head Up useful. PEU of Head Down and Head Up were different from the scale mean (*p* = 0.002; *p* = 0.001, respectively), indicating that both ADAS were considered easy to use. Moreover, while IU of Head Down was significantly different from the scale mean (*p* = 0.031), the IU of Head Up was not different from the scale mean (*p* = 0.659). Younger drivers did not intend to use Head Down, while they remained undecided regarding the Head Up.

The comparison of youth scores for each ADAS on the three variables showed that PU and PEU of Head Up were significantly higher than those of Head Down (*p* = 0.003, *p* = 0.003) (see Supplementary Table S2). Thus, younger drivers found Head Up more useful and easier to use than Head Down. In contrast, no significant difference (*p* = 0.069) was identified for IU.

#### 3.8.2. Older drivers

The results showed that the two ADAS were rather well accepted by older drivers. As with younger drivers, the PU of Head Down (*p* = 0.007) and Head Up (*p* = 0.001), the PEU of Head Down (*p* = 0.001) and Head Up (*p* = 0.001), and the IU of Head Down (*p* = 0.013) and Head Up (*p* = 0.010) were significantly different from the scale mean (see Supplementary Table S3) indicating that older drivers found both ADAS useful and easy to use, and that they intended to use them.

The results of the paired sample t-tests showed no significant difference in the older drivers between the PU, PEU and IU of the Head Down and Head Up (all ps > 0.225) (see Supplementary Table S4). This means that older people have the same level of acceptance for Head Down and Head Up.

#### 3.8.3. Comparison younger vs. older drivers

##### 3.8.3.1. Head Down

**Figure 14:**
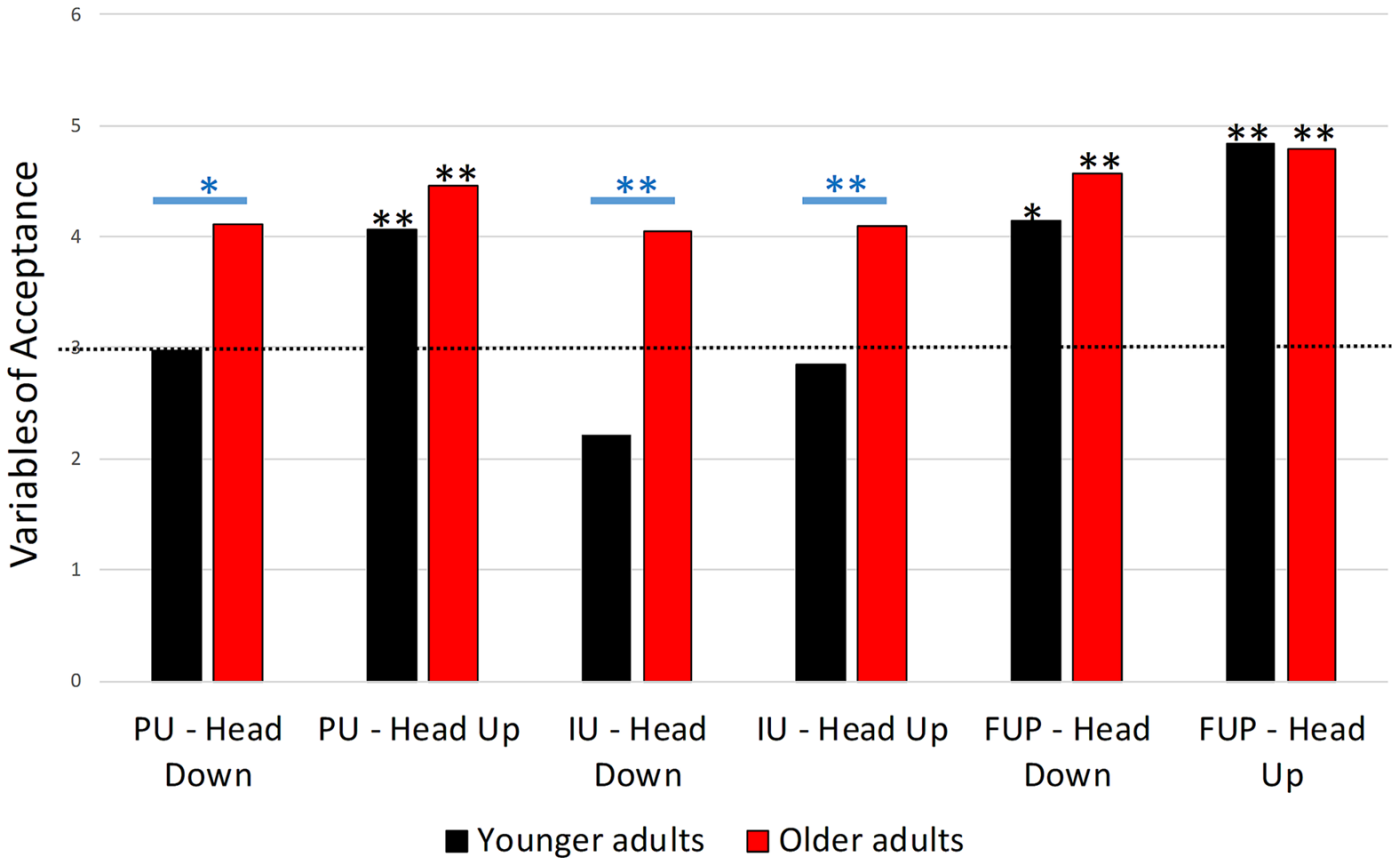
Graphical representation of young and older drivers’ acceptance scores for Head Down and Head Up.

The PU of Head Down among older drivers was significantly higher than that of younger drivers (*p* = 0.040) (see Supplementary Table S5), as was the IU (*p* = 0.001) (Figure 14). These results indicated that older drivers find Head Down more useful than young drivers. No significant difference was identified between older and younger drivers for the PEU (*p* = 0.311).

##### 3.8.3.2. Head Up

No significant differences were identified between older and younger for Head Up PU and PEU (*p* = 0.244; *p* = 0.722) (Figure 14). In contrast, older drivers were significantly more likely to intend to use Head Up than younger drivers (*p* = 0.016).

## 4. Discussion

The objective of this study was to determine to what extent driver assistance systems (ADAS) can help older drivers negotiate intersection crossing. To this end, we compared the regulation behavior (performance, displacement kinematics and visual strategies) produced by both young and older drivers when intercepting a moving inter-vehicular interval depending on whether or not they benefited from a driving assistance system. More precisely, the effectiveness and acceptance of two specific ADAS have been tested which allowed concurrent feedback to be displayed either on the dashboard (Head Down) or directly on the approaching traffic flow (Head Up). The results revealed strong similarities in the behavioral regulations that allow drivers to cross an intersection safely with or without the use of an ADAS regardless of the population. The information-taking strategies are strongly impacted by the type of ADAS. Finally, the elderly had a good acceptance of ADAS (significantly higher than the mean of the Likert scale), unlike young drivers.

### 4.1. Analysis of the regulations

As a reminder, in order to induce displacement speed regulations, the initial distance between the participants and the intersection was manipulated, giving rise to five Offsets. An offset requires appropriate deceleration (Offsets 1 and 2) or acceleration (Offsets −1 and − 2) to intercept the moving gap.

#### 4.1.1. No-ADAS

Both younger and older drivers produced displacement speed regulations that allowed the intersection to be crossed slightly before the center of the inter-vehicular interval, i.e., between the vehicle just ahead of the interval and the center of the interval, whatever the Offset condition. This result, consistent with our previous work, could reflect the use of a safe crossing strategy of leaving an optimal distance between one’s vehicle and the vehicles delimiting the inter-vehicular space to be crossed (Louveton et al., 2012a,b, 2018; Mathieu et al., 2017a,b; Tran Van et al., 2022). The results also reveal early and gradual displacement speed regulations throughout the approach, allowing participants (both young and old) to compensate for any initial offset. These displacement speed regulations are functional as they result in a gradual convergence of the current deviation towards the first half of the inter-vehicular space. Finally, comparative analysis of the patterns of variability in displacement speed and current deviation revealed compensatory variability (Bootsma and Van Wieringen, 1990; Bardy and Laurent, 1998; Chardenon et al., 2002; Camachon et al., 2007). An increase in displacement speed variability is accompanied by a decrease in the variability of the current deviation, results consistent with those obtained in our previous work (Louveton et al., 2012a,b, 2018; Mathieu et al., 2017a,b; Tran Van et al., 2022) but also with those described in different goal-directed tasks (Bootsma and Van Wieringen, 1990; Michaels and Oudejans, 1992; Bardy and Laurent, 1998). Taken as a whole, they illustrate the functional nature of the relationships linking perceptual and motor sides during the completion of the task (Gibson, 1979; Michaels and Carello, 1981; Warren, 2006).

#### 4.1.2. Head Down

As in the No-ADAS condition, early and gradual speed changes were observed in Head Down condition, giving rise to a progressive convergence of the current deviation towards the target crossing zone. Compensatory variability also appeared, with a gradual increase in speed variability throughout the approach giving rise, during the second part of the approach, to a decrease in current deviation variability. These results highlight that adding a driving assistance system (Head Down) does not interfere with the implementation of control mechanisms based on functional relationships between perception and action. They also revealed several specificities in comparison with the No-ADAS condition. In the two negative Offset conditions the participants tend to cross the intersection in the second half of the inter-vehicular gap (see Figure 7). Consequently, the safety margin while crossing the interval is slightly reduced in Head Down condition in comparison with the No-ADAS condition. While we thought that the presence of augmented information would lead the participants to produce safer behavior, the opposite is happening. This is even more true for older drivers (*cf.*, Figure 9). Relatively similar observations were made by Dotzauer et al. (2013). This result is compatible with the Risk Homeostasis Theory (RHT) of Wilde (1982). The RHT states that people have a target level of risk and will modify their behavior, in order to maintain this. Thus, it is conceivable that the accurate representation of the future crossing location thanks to Head Down reduces the level of risk perceived by the participants, so that they do not feel the need to target the first half of the inter-vehicular space.

Another specificity in the displacement kinematics in Head Down condition in comparison with the No-ADAS condition lies in the decrease in the overall variability of both displacement speed and current deviation. It is worth mentioning that the decrease in overall behavioral variability does not affect the presence of compensatory variability. It may be thought that the augmented information provided by Head Down makes it possible to remove part of the uncertainty associated with the regulations to be produced to perform the crossing task.

#### 4.1.3. Head Up

Taken as a whole, the results collected in Head Up condition are comparable to those obtained in the Head Down condition. Once again compensatory variability is present so that it is possible to claim that the informational augmentation contained in the ADAS has made it possible to preserve the natural link between perception and action.

The same particularities as those mentioned in the previous section (Head Down) appear when comparing the regulation behavior produced without ADAS and with Head Up. In the Offset −2 condition, the crossing is even more staggered in the second half of the inter-vehicular interval (*cf.*, Figure 7). An explicit materialization of the future crossing location does not encourage participants to target the first half of the interval, notably for older participants. As already observed with Head Down, the overall kinematics variability is lower in comparison with the variability observed in the No-ADAS condition. This decrease in overall variability combined with the preservation of compensatory variability is particularly interesting because it reveals that the ADAS constitutes a perceptual aid which is not intended to replace the control mechanisms implemented in its absence.

### 4.2. Analysis of visual information pick-up strategies

#### 4.2.1. No-ADAS

The results revealed that the first part of the train of vehicle (SUV_Front) was the Area of Interest most looked at during a trial (between 55 and 65% of the total time spent in the AOIs). These results are comparable to those reported in our previous work (Tran Van et al., 2022) and no significant differences were observed between older and younger drivers. As the drivers approached the intersection, they were aiming for the first part of the inter-vehicular space. This would explain the importance of the information located on the vehicle train preceding the inter-vehicular space to be crossed, to control the approach speed. Analysis also revealed differences in the information detection strategies implemented as a function of Offset. For negative offset (−2, −1 s), the train of vehicles before the inter-vehicular space (SUV_Front) remains the information detection area primarily set by drivers, but the inter-vehicular space is also favored. These results reflect the impact of Offset on the information detection strategies implemented by the drivers.

#### 4.2.2. Head Down

The average percentage of time spent in each AOI as a function of dashboard condition revealed that the dashboard location (i.e., dashboard) was the most looked at, closely followed by the first part of the train of vehicles (SUV_Front). This analysis also revealed differences between younger and older drivers. In older drivers, the dashboard location (dashboard) is the Area of Interest most looked at during a trial (between 55 and 65% of the total time spent in the AOIs) whereas for younger drivers the dashboard location (dashboard) and the first part of the train (SUV_Front) are both gazed at between 30 and 40% of the time.

These results confirm that the concurrent feedback provided by the Head Down is used to perform the task when available. Two distinct strategies were used by younger and older drivers. Older drivers prioritized the information provided by the concurrent feedback to regulate their speed, a finding also reached by Dotzauer et al. (2013) when testing their ADAS. They associate it with a negative effect, as the driver tends to neglect other sources of information. This effect is not present in young drivers. The dual prioritization (dashboard) and the first part of the train (SUV_Front) exhibited by young drivers indicated that they used both the information provided by the dashboard and the location of the first part of the vehicle train to regulate their travel speed.

#### 4.2.3. Head Up

The average percentage of time spent in each AOI in the Head Up condition showed that inter-vehicular space is the area most looked by the drivers when approaching the intersection, closely followed by the first part of the train of vehicles (SUV_Front). This analysis also revealed differences between the Offsets. For negative Offsets and no Offset, inter-vehicular space is the area most looked at during a trial (between 50 and 60% of the total time spent on the AOIs) while for positive Offsets the first part of the vehicle train (SUV_Front) is the area looked at the longest. Therefore, the location of the augmented information at the beginning of the trial depends on the initial offset, i.e., for Offset −2 the cursor is first located in the last part of the vehicle train (SUV_Behind), while for Offset 2 the cursor is first located in the first part of the vehicle train (SUV_Front). These observations would seem to indicate that drivers rely primarily on the information given by the concurrent feedback to regulate their travel speed.

### 4.3. Acceptance

An analysis of the acceptance of ADAS after use was performed to ensure that drivers accepted ADAS designed to assist them in crossing intersections. Consistently with the literature on older drivers’ acceptance of ADAS (Son et al., 2015; Madigan et al., 2016), we find an age effect. Older drivers considered both ADAS useful and easy to use and intended to use them. Young drivers considered both ADAS to be easy to use, Head Down was rated as neither useful nor unnecessary and no intention of using it was collected. Head Up was considered useful, but younger drivers were undecided about whether to use it or not. This difference in usefulness and intention-to-use ratings between younger and older drivers is consistent with findings from previous studies that report that older drivers rated ADAS higher than younger drivers (Stevens, 2012). It could be explained by the original purpose of these ADAS. In fact, they were designed to help older drivers in situations where younger drivers do not face difficulties. This could also explain the result reported by the comparisons between our two populations, which revealed that older drivers found Head Down more useful than younger drivers and had more intention to use Head Up than younger drivers. Comparison of the two ADAS reveals that younger drivers found the Head Up more useful and easier to use than the Head Down, while older drivers reported no difference. This could be because older drivers will be more receptive to receiving help in any form. Motamedi and Wang (2017) showed that older drivers were motivated to use new technologies because of their realistic awareness of their driving abilities.

### 4.4. Limitation

The experimental task used in the present experiment is not an exact replica of a task encountered while driving in everyday life. Crossing without stopping through a continuous stream of vehicles on a cross street would be an illegal driving maneuver. Nevertheless, a number of driving tasks require displacement velocity adjustments to adapt to traffic flows (e.g., when entering a roundabout, when entering a freeway *via* an access ramp or when turning left with oncoming traffic). That’s the reason why, from our point of view, even if our experimental task has not been implemented to be representative of real-life tasks, it nonetheless requires a central skill in the context of driving a car: the ability to adjust one’s displacement speed in order to intercept a moving interval.

This study provides a baseline of information on how drivers react when they attempt to intercept a moving gap. However, it is possible to observe slight differences once the tests have been carried out in real-life conditions. These differences can be attributed to the fact that, in our simulations, the driver is in a controlled environment and knows that he is safe in the event of a collision. Future studies will therefore be carried out under real-life conditions to verify the validity of the results.

## 5. Conclusion

Our study revealed similarities in the behavioral regulations that allow drivers to safely cross an intersection with or without the use of an ADAS regardless of the population. The regulations produced were based on a close coupling between perception and action, resulting in functional speed adjustments throughout the approach. These results thus reveal that ADAS is a perceptual aid that is not intended to replace the control mechanisms implemented in the absence of ADAS. However, while we thought that the presence of augmented information would lead participants to adopt safer behavior, this was not really the case. It seems that both ADAS tested reassure the participants so that they no longer feel the need to target the first half of the inter-vehicular space. Analysis of the eye-tracking data allowed the identification of specific information-taking strategies according to the ADAS tested. The areas mainly looked at were, respectively, the areas where the ADAS was located and seem to confirm that drivers use our ADAS in part to gather information to regulate their approach to the intersection. Acceptance analysis reported a mixed acceptance of our ADAS by younger drivers and a good acceptance by older drivers. These results allow us to make a first approach to this perception aid that we wish to develop. They also allow us to provide improvement tracks. An ADAS that would be displayed at the driver’s request, i.e., when he/she considers it necessary, would probably better preserve drivers’ autonomy. The second interest would be to avoid creating a dependency on ADAS, which could lead to an inability to cross an intersection without ADAS in older drivers. The idea of a Head Up system is worth considering because, as shown by information-gathering strategies, it allows the driver to keep his/her eyes on the road. Conversely, the results underline that the development of ADAS based on Head Down information displays can be put aside. The strategies of information taking showed that it makes the elderly look away from the road more than half the time, which leaves little room for error.

## Data availability statement

The original contributions presented in the study are included in the article/Supplementary material, further inquiries can be directed to the corresponding author.

## Ethics statement

The studies involving humans were approved by Comité d’éthique pour la recherche en STAPS (CERSTAPS). The studies were conducted in accordance with the local legislation and institutional requirements. The participants provided their written informed consent to participate in this study.

## Author contributions

CB, JN, and GM are the supervisors of my PhD, and helped me to set up the reflection on the protocol, as well as the data processing and the writing of the article. NM is the expert who enabled us to carry out the measurements and data analysis specific to acceptance. CG is the engineer who contributed to the development of the device and data processing. All authors contributed to the article and approved the submitted version.

## Footnotes

^1^ www.erso.eu
