# Supplementary material for: Evaluation of assistance systems allowing older drivers to intercept moving inter-vehicular space
Source: Front Psychol. 2023 Oct 24;14:1244646. doi: 10.3389/fpsyg.2023.1244646 (PMC10629389; doi:10.3389/fpsyg.2023.1244646)
Supplement: Supplementary file 1 [file Table_1.docx]

Supplementary Material

Article Title

Table 1

Results of the one-sample *t*-test conducted on young drivers for the three acceptance variables (PU, PEU and IU) for the dashboard and embedded conditions.

| One-sample *t*-test | | | | |
| --- | --- | --- | --- | --- |
|  | **t** | **df** | **p** | Cohen’s d |
| PU-Head Down | -0.061 | 13 | .953 | 2.024 |
| PEU-Head Down | 3.809 | 13 | .002 | 3.691 |
| IU-Head Down | -2.416 | 13 | .031 | 1.820 |
| PU-Head Up | 4.778 | 13 | .001 | 4.853 |
| PEU-Head Up | 21.880 | 13 | .001 | 15.417 |
| IU-Head Up | -0.452 | 13 | .659 | 2.417 |

Table 2

Result of the paired samples *t-*test conducted on young drivers for the three acceptance variables (PU, PEU and IU) to compare the dashboard and embedded conditions.

| Paired samples *t-*test | | | | | |
| --- | --- | --- | --- | --- | --- |
| **Measure 1** | **Measure 2** | **t** | **df** | **p** | **Cohen’s d** |
| PU-Head Down | PU-Head Up | *-3.709* | *13* | *.003* | *-.991* |
| PEU-Head Down | PEU-Head Up | *-2.245* | *13* | *.043* | *-.6* |
| IU-Head Down | IU-Head Up | *-1.979* | *13* | *.069* | *-.529* |

Table 3

Result of the one-sample *t-*test conducted on older drivers for the three acceptance variables (PU, PEU and IU) for the dashboard and embedded conditions.

| One-sample *t*-test | | | | |
| --- | --- | --- | --- | --- |
|  | **t** | **df** | **p** | Cohen’s d |
| PU-Head Down | 3.177 | 13 | .007 | 3.125 |
| PEU-Head Down | 5.477 | 13 | .001 | 4.258 |
| IU-Head Down | 2.869 | 13 | .013 | 2.954 |
| PU-Head Up | 6.368 | 13 | .001 | 5.217 |
| PEU-Head Up | 17.419 | 13 | .001 | 12.477 |
| IU-Head Up | 3.010 | 13 | .010 | 3.008 |

Table 4

Result of the paired samples *t*-test conducted on older drivers for the three acceptance variables (PU, PEU and IU) to compare the dashboard and embedded conditions.

| Paired samples *t*-test | | | | | |
| --- | --- | --- | --- | --- | --- |
| **Measure 1** | **Measure 2** | **t** | **df** | **p** | **Cohen’s d** |
| PU-Head Down | PU-Head Up | -1.275 | 13 | .225 | -.341 |
| PEU-Head Down | PEU-Head Up | -.715 | 13 | .487 | -.191 |
| IU-Head Down | IU-Head Up | -.178 | 13 | .862 | -.047 |

Table 5

*t*-test result on young and old for the three acceptance variables (PU, PEU and IU) to compare the populations in the dashboard and embedded conditions.

|  | **t** | **df** | **p** | **Cohen’s d** |
| --- | --- | --- | --- | --- |
| PU-Head Down | -2.166 | 26 | .040 | -.819 |
| FUP-Head Down | -1.302 | 26 | .311 | -.390 |
| IU-Head Down | -3.743 | 26 | .001 | -1.415 |
| PU-Head Up | -1.191 | 26 | .244 | -.450 |
| FUP-Head Up | 0.360 | 26 | .722 | .136 |
| IU-Head Up | -2.570 | 26 | .016 | -.971 |
